# Comparison of trifocal or hybrid multifocal-extended depth of focus intraocular lenses: a systematic review and meta-analysis

**DOI:** 10.1038/s41598-021-86222-1

**Published:** 2021-03-23

**Authors:** Yueyang Zhong, Kai Wang, Xiaoning Yu, Xin Liu, Ke Yao

**Affiliations:** Eye Center of the Second Affiliated Hospital, Zhejiang University School of Medicine, No. 88 Jiefang Road, Hangzhou, 310009 China

**Keywords:** Medical research, Eye diseases

## Abstract

This meta-analysis aimed to evaluate the clinical outcomes following implantation of trifocal intraocular lenses (IOLs) or a hybrid multifocal-extended depth of focus (EDOF) IOL in cataract or refractive lens exchange surgeries. We examined 13 comparative studies with bilateral implantation of trifocal (898 eyes) or hybrid multifocal-EDOF (624 eyes) IOLs published through 1 March 2020. Better uncorrected and corrected near visual acuity (VA) were observed in the trifocal group (MD: − 0.143, 95% CI: − 0.192 to − 0.010, *P* < 0.001 and MD: − 0.149, 95% CI: − 0.217 to − 0.082, *P* < 0.001, respectively), while the hybrid multifocal-EDOF group presented better uncorrected intermediate VA (MD: 0.055, 95% CI: 0.016 to 0.093, *P* = 0.005). Trifocal IOLs were more likely to achieve spectacle independence at near distance (RR: 1.103, 95% CI: 1.036 to 1.152, *P* = 0.002). The halo photic effect was generated more frequently by the trifocal IOLs (RR: 1.318, 95% CI: 1.025 to 1.696, *P* = 0.031). Contrast sensitivity and subjective visual quality yielded comparable results between groups. Trifocal IOLs demonstrated better performance at near distance but apparently led to more photic disturbances. Our findings provided the most up-to-date and comprehensive evidence by comparing the benefits of advanced IOLs in clinical practice.

## Introduction

The ultimate goal of cataract treatment, presbyopia correction and refractive lens exchange (RLE) surgery is to reduce dependence on spectacles and attain full range of vision from near to far distances^1^. With modern techniques and pharmaceutical recommendations, intraocular lenses (IOLs) implantation embraces the advantages of effective visual rehabilitation, reduced risk of complications and low economic burden. Consequently, increasing demands for high-quality vision at all distances and spectacle independence have prompted the development of various advanced multifocal IOLs designs since 1980s^2^.

Trifocal IOLs, which work by splitting lights into three different foci, provides comparable visual acuities (VAs) at all distances to bifocal IOLs^3^. The superiority of trifocal IOLs over monofocal and bifocal IOLs with respect to better near and intermediate VAs and less photic phenomena has been supported by various controlled clinical studies and meta-analyses^4–6^. Initial studies concerning the visual outcomes of trifocal IOLs, such as the FineVision Micro F IOL (PhysIOL Liege, Belgium) and the AT LISA tri 839MP IOL (Carl Zeiss Meditec AG, Jena, Germany) have demonstrated promising results^3,7–13^. Another trifocal IOL design, the AcrySof IQ PanOptix IOL (Alcon Surgical, Inc., Fort Worth, TX), which diffracts the light from the first focal point to the distance focus, achieves a more natural transition from intermediate to far distance with improved visual outcomes^14^. However, due to their design properties, all trifocal IOLs inevitably reduce contrast sensitivity and generate photic effects^3^.

As one of the frequently used multifocal IOLs, TECNIS Symfony ZXR00 (Johnson & Johnson Vision, Santa Ana, California, USA) is based on a proprietary echelette design and achromatic technology^15^. Such features, along with negative spherical aberration correction, have been shown to provide a significantly improved retinal image quality with minimal optical side effects^15–17^. However, due to some commercially biased information, most of the existing studies have defined Symfony as an extended depth of focus (EDOF) IOL, which in fact is a hybrid multifocal-EDOF IOL^18^. The so-called EDOF effect of Symfony is mainly attributed to its multifocality and echelette design^16^. Therefore, recognition and clarification of this novel issue is of significance for ophthalmologists to better understand the design and features of TECNIS Symfony.

Although several prospective trials have compared the clinical outcomes of trifocal and the hybrid multifocal-EDOF IOLs in recent years, there is still debate on which type of IOLs could offer the maximum benefits^4,13,19–25^. Furthermore, while a previous meta-analysis relied on a small sample size and inadequate parameters^26^, in the present study, we included data from populations in Europe, Asia and South America and stratified the data according to the different types of IOL. Therefore, we have conducted an up-to-date and comprehensive meta-analysis of the existing randomized controlled trials (RCTs) and prospective cohorts to compare the visual performance, spectacle independence and photic disturbance of trifocal IOLs and the hybrid multifocal-EDOF IOL after cataract or RLE surgeries.

## Results

### Search process

**Figure 1:**
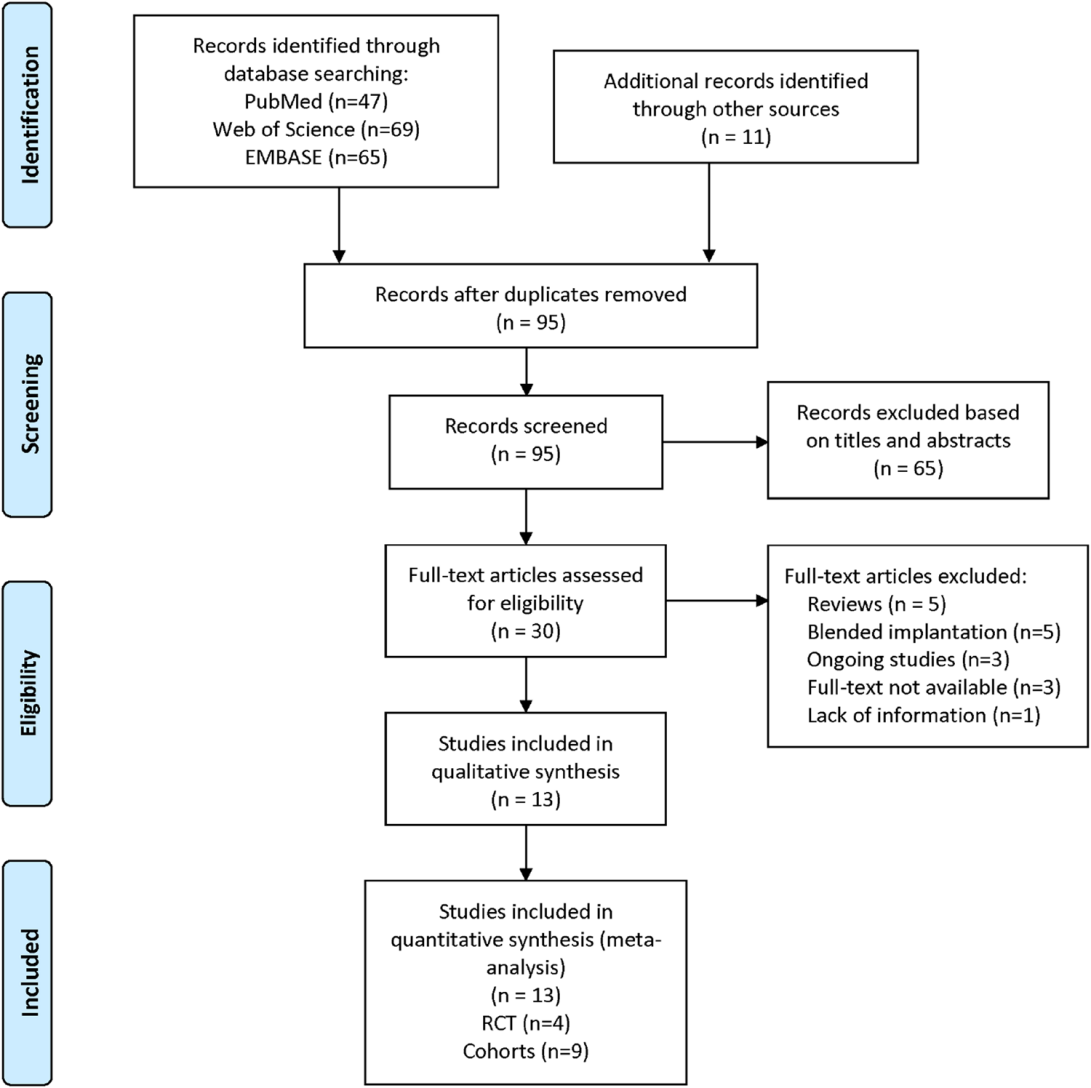
Flow chart depicting selection of studies. RCTs, randomized controlled trials.

Of the 192 articles identified (47 from PubMed, 69 from Web of Science and 65 from EMBASE), we excluded 97 duplicates, and 65 articles were excluded based on their titles and abstracts. Full-text assessment consisted of 30 articles, of which 17 articles were excluded for the following reasons: five were reviews, five were conducted with blended implantation, three were ongoing studies, and four did not provided full-text or adequate information. Ultimately, four RCTs^7,20,22,27^ and nine non-randomized comparative studies (NRCSs)^4,11,13,19,21,23–25,28^ were included in the current meta-analysis. Figure 1 presents the flow diagram of the selection process.

### Study characteristics

Table 1 summarizes the descriptive characteristics and quality assessment of each study. All studies were published from August 2016 through March 2020, with follow up duration ranging from 1 to 28 months. 10 of the included studies were performed in Europe, two were in Asia and one in South America. In total, 13 studies which reported on 898 eyes (449 patients) implanted with trifocal IOLs and 624 eyes (312 patients) implanted with the hybrid multifocal-EDOF IOL were included in the meta-analysis. The average Jaded scale for the four RCTs was 3.8 points and average Newcastle–Ottawa Scale (NOS) for the NRCSs was 7.6 points (Supplementary Tables S1 and S2).

**Table 1:** Characteristics of the included studies comparing trifocal and the hybrid multifocal-extended depth of focus IOLs (n = 13).

Author, year	Study design	Location	Follow-up (months)	IOL types	Patients (n)	Eyes (n)	Age (years)	Gender (male/female)	Quality Score
Hamid and Sokwala, 2016^11^	NRCS	UK	6	FineVision	50	100	58.2 ± 9.4	17/33	8
				AT LISA tri 839MP	50	100	56.9 ± 7.0	17/33	
				Symfony	50	100	57.8 ± 6.0	17/33	
Monaco, 2017^22^	RCT	Italy	4	PanOptix	20	40	66.0 ± 5.5	11/9	4^a^
				Symfony	20	40	67.0 ± 8.5	9/11	
Ruiz-Mesa, 2017^24^	NRCS	Spain	12	FineVision	20	40	54.5 ± 7.2	9/11	8
				Symfony	20	40	59.5 ± 8.9	8/12	
Cochener, 2018^7^	RCT	France	6	PanOptix	20	40	62.5 ± 4.6	NR	2^a^
				FineVision	20	40	62.5 ± 4.6	NR	
				Symfony	20	40	69.2 ± 8.4	NR	
Escandón-García, 2018^19^	NRCS	Portugal	1–3	PanOptix	7	14	62.3 ± 9.0	1/6	7
				FineVision	23	46	62.6 ± 8.0	7/16	
				Symfony	15	30	63.5 ± 9.4	2/13	
Mencucci, 2018^21^	NRCS	Italy	3	PanOptix	20	40	70.1 ± 4.8	NR	8
				AT LISA tri 839MP	20	40	71.6 ± 4.4	NR	
				Symfony	20	40	68.9 ± 4.8	NR	
Ruiz-Mesa, 2018^25^	NRCS	Spain	8–28	PanOptix	20	40	63.8 ± 8.1	14/6	8
				Symfony	14	28	63.1 ± 10.0	5/9	
Böhm, 2019^13^	NRCS	Germany	3	PanOptix	27	54	63.4 ± 8.9	13/14	7
				AT LISA tri 839MP	27	54	63.5 ± 7.9	10/17	
				Symfony	26	52	69.2 ± 8.2	18/8	
de Medeiros, 2019^4^	NRCS	Brazil	6–12	PanOptix	13	26	NR	(12/14)^b^	8
				Symfony	13	26	NR		
Rodov, 2019^23^	NRCS	Israel	1	FineVision	50	100	67.0 ± 6.7	14/36	6
				Symfony	50	100	67.2 ± 9.8	26/24	
Singh, 2019^28^	NRCS	India	6	FineVision	20	40	66.1 ± 5.1	8/12	8
				Symfony	20	40	69.1 ± 6.1	9/11	
Gil, 2020^20^	RCT	Spain	6	AT LISA tri 839MP	10	19	68.7 ± 10.3	2/8	4^a^
				Symfony	10	20	68.2 ± 6.2	3/8	
Webers, 2020^27^	RCT	Netherlands	3	AT LISA tri 839MP	13	26	70.4 ± 6.1	6/7	5^a^
				Symfony	14	28	67.6 ± 12.2	4/10	

IOL, intraocular lens; RCT, randomized controlled trial; NRCS, non-randomized controlled study; NR, not reported.

^a^RCTs assessed with the Jaded Scale.

^b^The ratio for both groups, no separate data provided.

### Primary outcomes

#### Visual acuity

**Figure 2:**
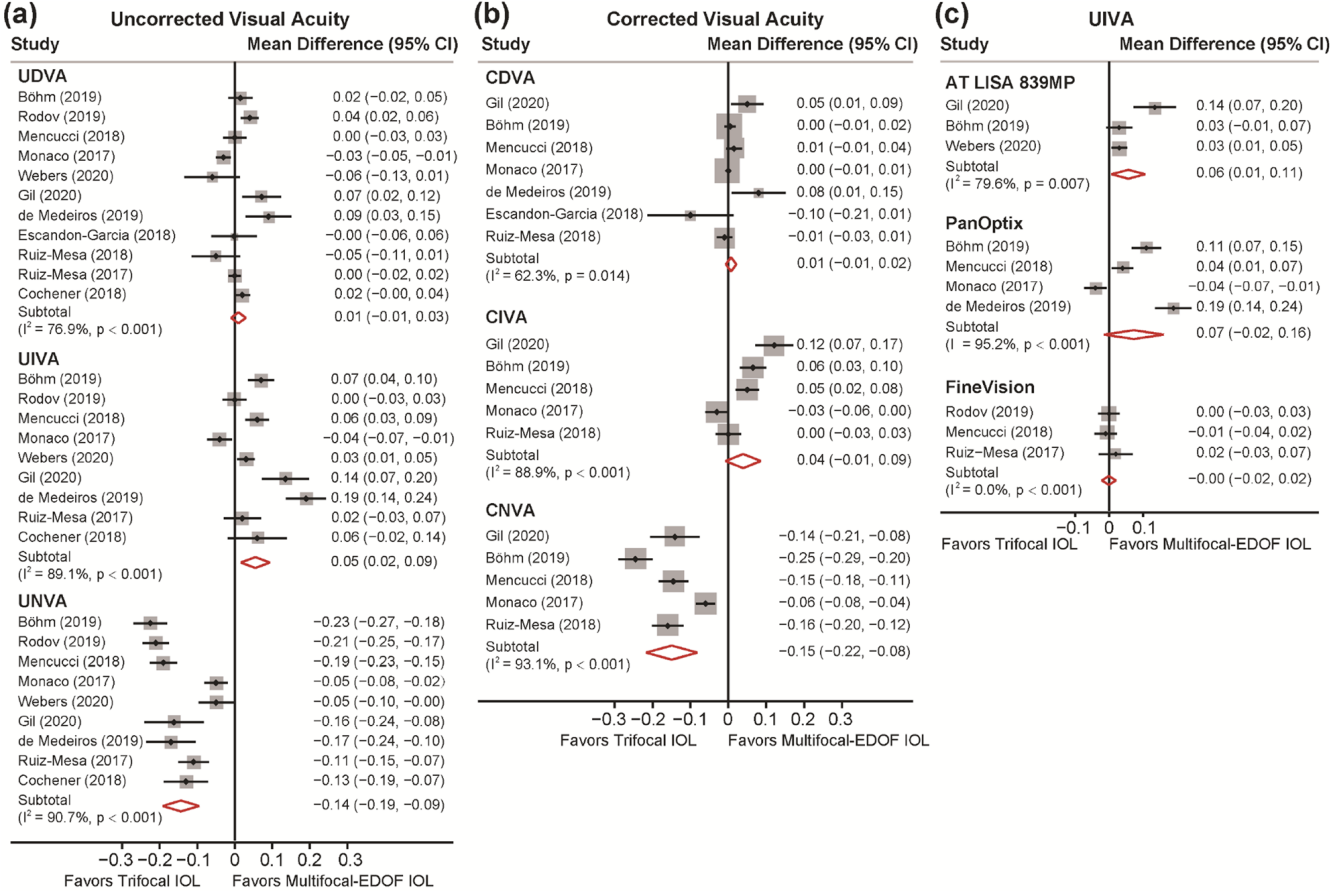
Visual acuities (VAs) in logMAR comparing trifocal and the hybrid multifocal-extended depth of focus (EDOF)intraocular lens (IOL). (**a**) Forest plot displaying mean difference (MD) of uncorrected VA of far, intermediate and near distance. (**b**) Forest plot displaying MD of corrected VA of far, intermediate and near distance. (**c**) Forest plot displaying MD of uncorrected intermediate VA comparing three types of trifocal IOLs and the hybrid multifocal-EDOF IOL.

12 studies reported data on uncorrected and corrected distance VA (UDVA and CDVA), uncorrected and corrected intermediate VA (UIVA and CIVA) and uncorrected and corrected near VA (UNVA and CNVA) (Fig. 2a,b). The analyses did not reveal significant differences in UDVA and CDVA between trifocal IOLs and the hybrid multifocal-EDOF IOL (MD: 0.010, 95% CI: − 0.010 to 0.030, *P* = 0.334 for UDVA; MD: 0.007, 95% CI: − 0.007 to 0.021, *P* = 0.307 for CDVA). In terms of intermediate visual performance, the hybrid multifocal-EDOF IOL provided better UIVA (MD: 0.055, 95% CI: 0.016 to 0.093, *P* = 0.005) and comparable CIVA (MD: 0.039, 95% CI: − 0.008 to 0.086, *P* = 0.101) with trifocal IOLs. In addition, the trifocal group presented significantly better results of UNVA (MD: − 0.143, 95% CI: − 0.192 to − 0.010, *P* < 0.001) and CNVA (MD: − 0.149, 95% CI: − 0.217 to − 0.082, *P* < 0.001). To determine the possible source of the high between-study heterogeneity, sensitivity analyses were conducted by omitting one study at a time (Supplementary Tables S3 and S4). The outcomes did not substantially alter the significance of the pooled estimate, confirming the stability of the results.

Considering the various characteristics of trifocal IOLs, a subgroup analysis was performed, and the data were stratified according to the type of IOL implanted (Fig. 2c and Supplementary Fig. S1). Respectively, six, four and three studies reported on PanOptix, FineVision and AT LISA tri 839MP IOLs. All three IOLs all presented comparable UDVA and significantly better UNVA when compared to the hybrid multifocal-EDOF IOL. However, AT LISA tri 839MP IOLs performed worse in UIVA (MD: 0.951, 95% CI: 0.062 to 1.839, *P* = 0.036), while such discrepancy was not observed in the other two trifocal IOLs.

#### Refraction

**Figure 3:**
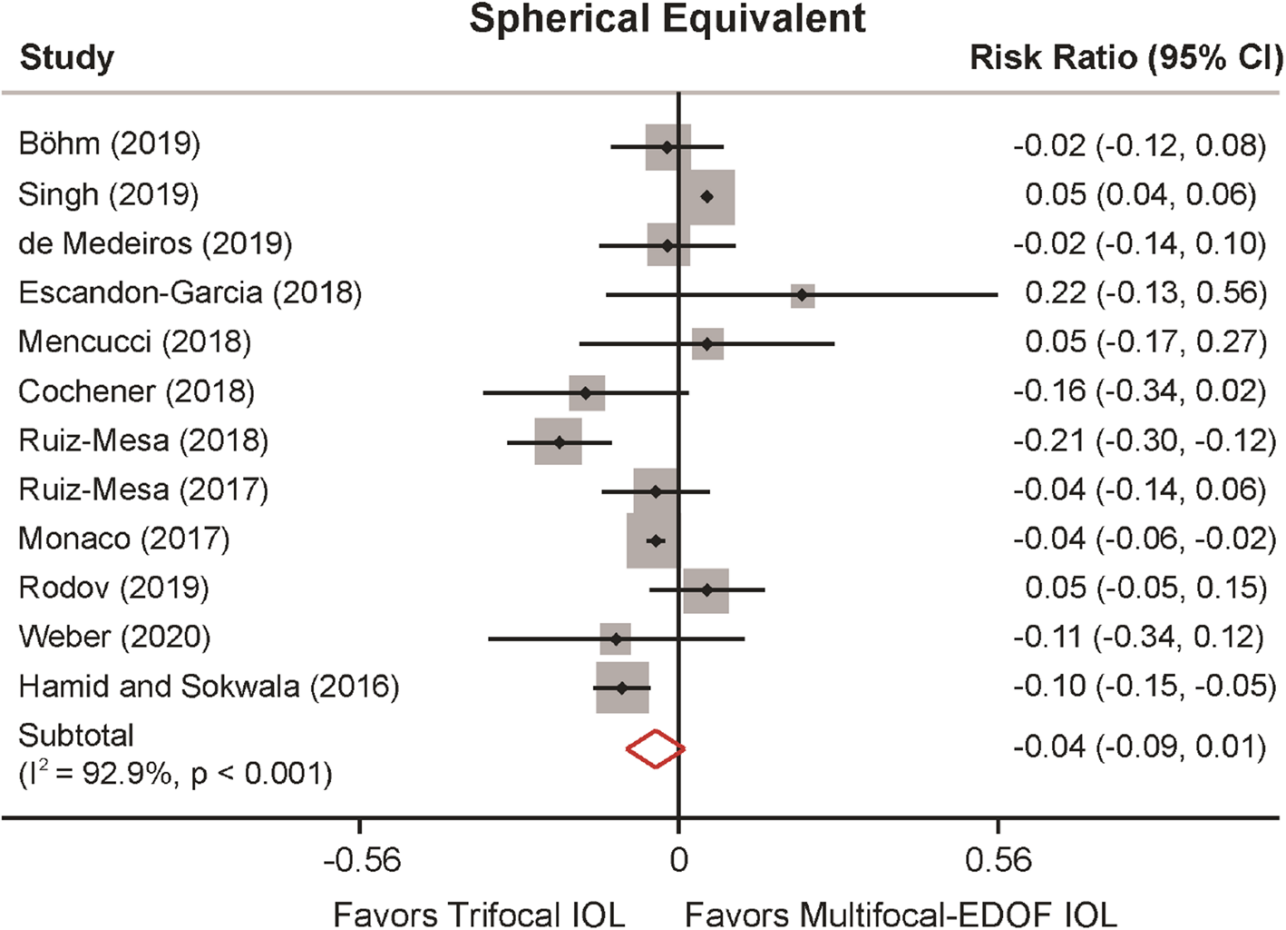
Spherical equivalent comparing trifocal and the hybrid multifocal-extended depth of focus (EDOF) intraocular lens (IOL).

The postoperative spherical equivalent was recorded in 12 studies (Fig. 3). Our pooled results did not observe a significant difference in spherical equivalent between the trifocal and the hybrid multifocal-EDOF groups (MD: − 0.040, 95% CI: − 0.092 to 0.011, *P* = 0.121). The studies were characterized by high heterogeneity (*I*^2^ = 92.9%, *P* = 0.005). Nevertheless, after excluding Singh et al*.*^28^, heterogeneity decreased (from 92.9 to 61.8%), and spherical equivalent results revealed significantly better performance in the trifocal group than the hybrid multifocal-EDOF group (MD: − 0.057, 95% CI: − 0.101 to − 0.013, *P* = 0.011) (Supplementary Table S5 and Fig. S3). Meta-regression analysis indicated an association between spherical equivalent and follow-up duration (*P* = 0.037) and explained 39.75% of the heterogeneity (Supplementary Fig. S4).

#### Spectacle independence

**Figure 4:**
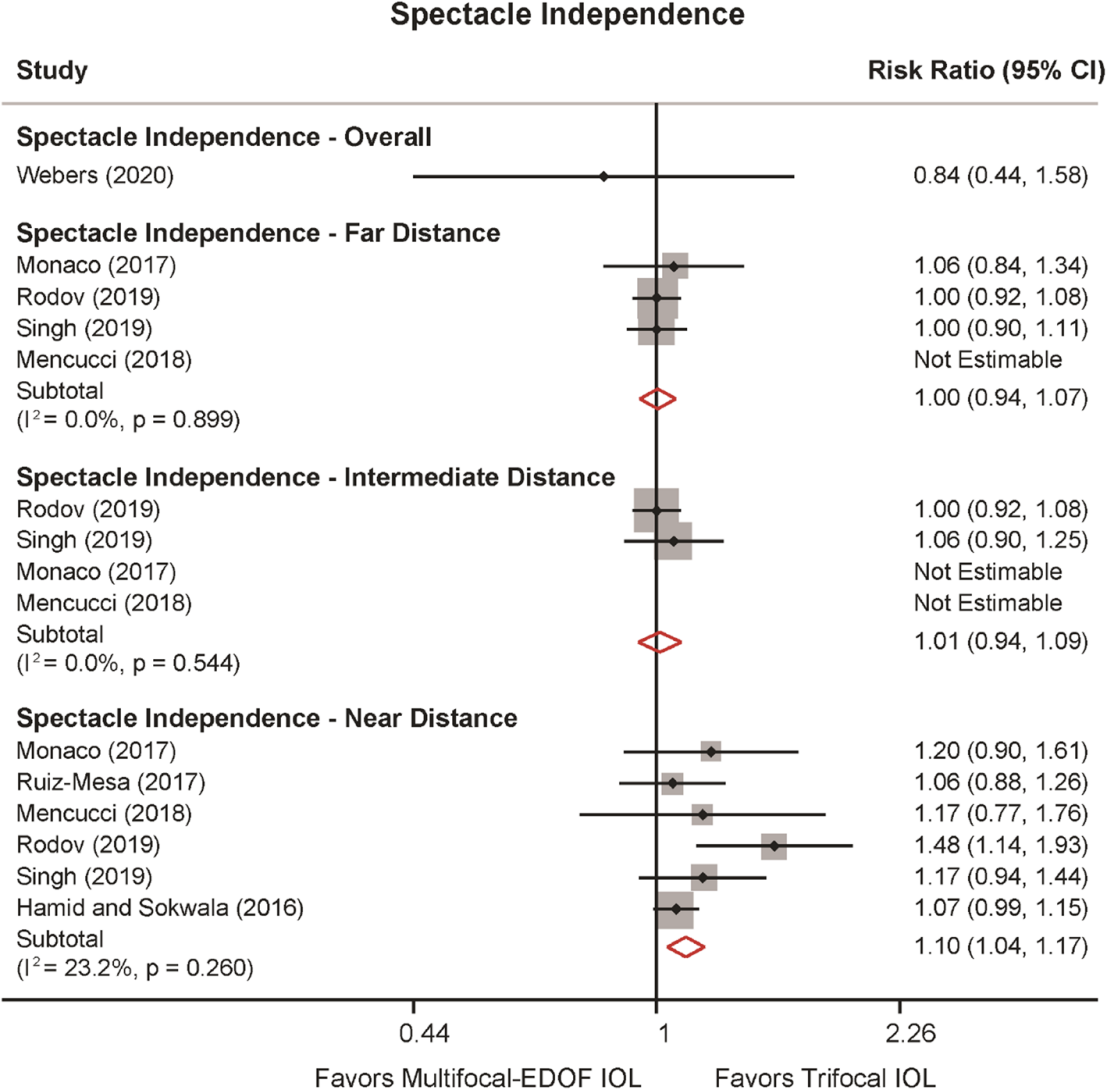
Spectacle independence of far, intermediate and near distance comparing trifocal and the hybrid multifocal-extended depth of focus (EDOF) intraocular lens (IOL).

Seven studies provided data for spectacle independence of far, intermediate and near distances (Fig. 4). At far and intermediate distances, spectacle independence did not reveal significant differences between the two groups. However, trifocal IOLs were 10% more likely to achieve spectacle independence at near distance (RR: 1.103, 95% CI: 1.036 to 1.152, *P* = 0.002). No heterogeneity was detected in spectacle independence for far and intermediate distances (*I*^2^ = 0%), and low heterogeneity was detected in spectacle independence for near distance (*I*^2^ = 23.2%,* P* = 0.260).

#### Photic disturbances

**Figure 5:**
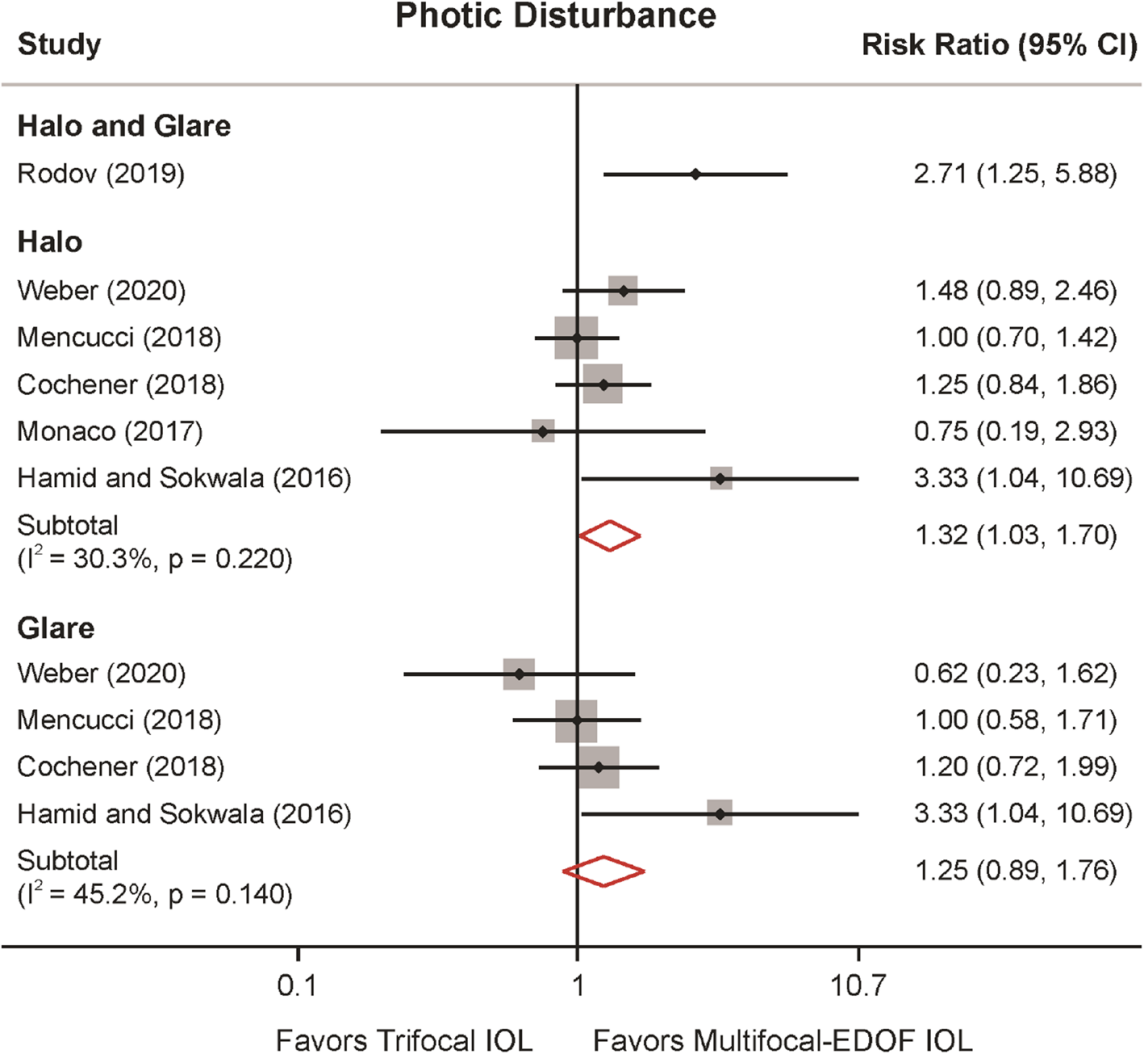
Photic disturbance of halo and glare comparing trifocal and the hybrid multifocal-extended depth of focus (EDOF) intraocular lens (IOL).

In most of the studies, the presence of halos and glare has been subjectively evaluated without a numerical or consistent grading system. For this reason, the outcomes reported and the conclusions of the authors are somehow inconsistent or not properly reported by adequate methodology. Photic disturbances namely halo and glare effects were discussed in seven studies (Fig. 5). Rodov et al*.*^23^ reported halo or glare symptoms altogether and was analyzed separately. They claimed that trifocal IOLs (FineVision) were more likely to cause photic disturbances. Our results suggested that trifocal IOLs were 32% more likely to generate a halo effect (RR: 1.318, 95% CI: 1.025 to 1.696, *P* = 0.031). Furthermore, although three studies observed fewer glare disturbance in the hybrid multifocal-EDOF IOL, the results did not reveal significance (RR: 1.251, 95% CI: 0.889 to 1.761, *P* = 0.198). Moderate heterogeneity was found in the halo and glare effects (*I*^2^ = 30.3%,* P* = 0.220, and *I*^2^ = 45.2%,* P* = 0.140, respectively).

### Secondary outcomes

#### Defocus curves

11 studies reported on defocus curves, and the descriptive information were presented in Table 2. Most studies demonstrated that trifocal IOLs outperformed the hybrid multifocal-EDOF IOL from − 4D to − 2.5D (22 to 40 cm) under photopic condition. However, results at intermediate distance (66 to 100 cm) were inconsistent in different types of trifocal IOLs. For example, for AT LISA tri 839MP IOL, three studies reported inferior performance than the hybrid multifocal-EDOF IOL from -1.5D to -1D, while Webers et al*.* reported no significant differences^11,13,20,27^. Two studies found FineVision IOL to be worse than the Symfony IOL at intermediate distance and two did not reveal significant differences^7,11,19,24^. For PanOptix IOL, Monaco et al*.* observed better performance than Symfony^22^. On the other hand, three studies observed worse performance at intermediate distance, and two of no significant differences^4,7,13,19,25^.According to Cochener et al*.*^7^, slight humps were noticed at the principle foci in trifocal IOLs, whereas the hybrid multifocal-EDOF IOL presented a smoother curve in the shape of a dome, indicating a stable and continuous visual performance.

**Table 2:** Descriptive information of defocus curve.

Author, year	Trifocal IOLs	The Hybrid Multifocal-EDOF IOL	Results of defocus curve
Hamid and Sokwala, 2016^11^	FineVision AT LISA tri 839MP	Symfony	Trifocal IOL worse than Symfony at -1D and -1.5D
Monaco, 2017^22^	PanOptix	Symfony	Trifocal IOL better than Symfony at -1.5 D and from -4D to -2.5D
Ruiz-Mesa, 2017^24^	FineVision	Symfony	Trifocal IOL better than Symfony from -4D to -2.5D
Cochener, 2018^7^	PanOptix FineVision	Symfony	Trifocal IOL had slight humps at the principal foci, Symfony had smoother curve in the shape of a dome
Escandón-García, 2018^19^	PanOptix FineVision	Symfony	Trifocal IOL better than Symfony at -2.5D and -3D, trifocal IOL worse than Symfony at -1D
Ruiz-Mesa, 2018^25^	PanOptix	Symfony	Trifocal IOL better than Symfony from -4D to -2D
Böhm, 2019^13^	PanOptix AT LISA tri 839MP	Symfony	Trifocal IOL better than Symfony at -2.5D, AT LISA tri 839MP worse than Symfony from -2D to -1D, PanOptix worse than Symfony at -1D
de Medeiros, 2019^4^	PanOptix	Symfony	Trifocal IOL better than Symfony from -5D to -2D, trifocal IOL worse than Symfony from -1D to 0D
Gil, 2020^20^	AT LISA tri 839MP	Symfony	Trifocal IOL better than Symfony from -4.5D to -2D, trifocal IOL worse than Symfony from -1.5D to 0D
Webers, 2020^27^	AT LISA tri 839MP	Symfony	Trifocal IOL better than Symfony from -4D to -2.5D

EDOF, extended depth of focus; IOL, intraocular lens.

#### Contrast sensitivity

Among nine studies that assessed contrast sensitivity (CS), seven reported comparable results in the two groups^7,19,22,24,25,27,28^ (Table 3). Hamid and Sokwala^11^ and Mencucci et al*.*^21^ claimed that the hybrid multifocal-EDOF IOL provided significantly higher values of CS values than trifocal IOLs under photopic and scotopic conditions. Escandón-García et al*.*^19^ found that the hybrid multifocal-EDOF IOL performed better at a frequency of 1.5 cycles per degree (cpd) under scotopic conditions.

**Table 3:** Descriptive information of contrast sensitivity.

Author, year	Trifocal IOLs	The Hybrid Multifocal-EDOF IOL	Results of contrast sensitivity
Hamid and Sokwala, 2016^11^	FineVision AT LISA tri 839MP	Symfony	Significantly better in Symfony IOL in both photopic and scotopic conditions
Monaco, 2017^22^	PanOptix	Symfony	No statistically significant differences in both photopic and scotopic conditions
Ruiz-Mesa, 2017^24^	FineVision	Symfony	No statistically significant differences and both below the normal range at 12 cpd and 18 cpd in photopic and scotopic conditions
Ruiz-Mesa, 2018^25^	PanOptix	Symfony	No statistically significant differences and both below the normal range at 12 cpd and 18 cpd in photopic and scotopic conditions
Cochener, 2018^7^	PanOptix FineVision	Symfony	No statistically significant differences in both photopic and scotopic conditions
Mencucci, 2018^21^	PanOptix AT LISA tri 839MP	Symfony	Significantly better in Symfony IOL in both photopic and scotopic conditions
Escandón-García, 2018^19^	PanOptix FineVision	Symfony	Significantly better in Symfony IOL at 1.5 cpd in scotopic conditions
Singh, 2019^28^	FineVision	Symfony	No statistically significant differences in both photopic and scotopic conditions
Webers, 2020^27^	AT LISA tri 839MP	Symfony	No statistically significant differences in both photopic and scotopic conditions

EDOF, extended depth of focus; IOL, intraocular lens; cpd, cycles per degree.

#### Visual quality and satisfaction

Quality of vision and satisfaction were evaluated with validated questionnaires as presented in Supplementary Tables S6 and S7, respectively. In general, a high percentage of satisfaction was reported in each group^7,11,21,27^. Subjective visual quality was assessed with different questionnaires, and most studies presented comparable results^4,7,22,27,28^. However, Gil et al*.*^20^ suggested the hybrid multifocal-EDOF IOL presented better performance in all subscales, while performed worse on the bothersome subscale according to Escandón-García et al*.*^19^.

#### Publication bias

Publication bias was tested using Egger linear regression test and the Begg rank correlation test (Supplementary Table S8). The results did not show significant bias in any of the comparisons, which was consistent with the funnel plots (Supplementary Fig. S5).

## Discussion

The present systematic review and meta-analysis investigated clinical outcomes after implantation of trifocal IOLs and the hybrid multifocal-EDOF IOL, providing the most up-to-date evidence for selecting the proper IOLs for patients. With regard to VAs, high-quality evidence was found that trifocal IOLs presented significantly better UNVA and CNVA than the hybrid multifocal-EDOF IOL, while the hybrid multifocal-EDOF group showed better results in UIVA. In addition, patients in the trifocal group were more likely to achieve spectacle independence at near distance; however, they also had a greater potential to develop photic effects, such as halo and glare.

In the recent development of presbyopia correcting IOLs, pure EDOF IOL, which creates an elongated focal point to enhance depth of focus, has been a major topic of interest. Nevertheless, the term was frequently confused with multifocality concepts that abundant studies have regarded Symfony as a so-called EDOF IOL^16^. According to recent publications, Tecnis Symfony is by design a hybrid multifocal-EDOF IOL with achromatic diffractive echelette features^16,18^. Instead of elongating the focal point, Symfony achieves such effects mainly because of its multifocality. Consequently, it is crucial to clarify the biased definition and raise awareness among ophthalmologists to better understand the features of the widely used Symfony IOL.

Trifocal IOLs, with various designs and technologies, have been developed to compensate for the impaired vision of monofocal IOLs at near and intermediate distances without compromising distance vision. To better evaluate the potential of IOL correction, assessments on VA alterations over a range of distances are required. In general, both IOL groups provided satisfactory vision across all distances. The combined results of the distance VAs of the included studies did not reveal significant differences, confirming that the advanced design of both IOLs were not detrimental to the distance focal point. The comparable performance of the two groups at distance region was also proved by the results from the defocus curves. Intermediate distance VA has been given heightened importance since most daily routine activities, such as driving, working and operating electronic devices, are performed at an intermediate distance. In the present meta-analysis, the hybrid multifocal-EDOF IOL yielded better UIVA than trifocal IOLs, which was not observed in the previous meta-analysis^26^. This finding was supported by the defocus curve in some studies, which showed that the hybrid multifocal-EDOF IOL performed better at the defocus level from -1.5D to -1D^4,11,13,19,20^. The different types of trifocal IOLs might be a source of discrepancies. For instance, the additional focal point for FineVision IOL was at 80 cm, while the focal point was at 60 cm for the other two trifocal IOLs. The subgroup analysis indicated that AT LISA tri 839MP performed worse at intermediate distance than the hybrid multifocal-EDOF IOL, contributing to the pooled results. However, inconsistent results among studies precluded definitive conclusion to extrapolate to all trifocal designed IOLs. Considering the limited study number and moderate between-study heterogeneity, our evidence of intermediate VA was insufficient to reach a definitive conclusion. Further research with appropriate distance at different focal points is warranted.

Near distance can be considered to be a comfortable reading distance. Due to its diffractive echelette design, the hybrid multifocal-EDOF IOL provides the best vision from far to intermediate distance with restraint of near vision^15^. Consistent with our findings in the defocus curves, all of the included studies demonstrated preferable uncorrected and corrected near VAs in the trifocal group over the hybrid multifocal-EDOF group, offering strong evidence and clinical significance. The relatively small values of VA (logarithm of the minimum angle of resolution, logMAR) values might contribute to the large variability of the data, which could explain the high heterogeneity. Further, the variation of the study design, VA assessment techniques and patients’ clinical conditions may have also contributed to the discrepancies among studies.

Refractive misses after IOL implantation leads to residual ametropia, reduced vision and dissatisfaction^29^. Our results on refractive outcomes demonstrated a slightly larger but not significant spherical equivalent in the hybrid multifocal-EDOF IOL, while such deviation did not negatively affect VAs or patient’s satisfaction. The lack of significance may be due to the inclusion of the study by Singh et al*.*^30^. According to Cochener et al*.*^7^, the achromatic echelette design of Symfony shows a greater tolerance of refractive error than the diffractive multifocal IOLs^31^. This characteristic and multifocality add to the hybrid multifocal-EDOF IOL’s effectiveness and stability in different clinical situations^32^. Previous studies and clinical experience implied that refractive data tended to remain stable 3 months after surgery^5^. Our meta regression analysis suggested that the discrepancies among the studies could possibly be related to follow-up time. Therefore, the outcome should be interpreted in the context of different follow-up durations and future studies with longer follow-up durations are required to assess the stability of the outcomes and occurrence of any complications.

Another aspect, which has been largely overlooked by research, concerns the patients’ eye care habits and quality of vision and life. Subjective benefits and spectacle independence over time are significant factors driving patients’ expectations^33^. Recently, a meta-analysis of multifocal IOLs found that approximately 80% of patients achieved spectacle independence after multifocal IOL implantation^34^. To offer a broader perspective, spectacle independence in our meta-analysis was measured across all distances. Based on strong evidence, more patients achieved spectacle independence at near distance in the trifocal group, which is in accordance with the better near VAs observed. As a subjective appraisal, spectacle dependence might be influenced by individual habits and lifestyles in real contexts; therefore, this conclusion should be interpreted with caution.

In terms of photic effects, any disturbance of the light through the optic axial would lead to subjective misperception and various kinds of photopsia, among which halo and glare effects were reported to occur the most frequently. Nonconformities on the optical path, such as cataracts or multifocal IOL implantations may contribute to such disturbances^15^. Up to 90% of patients reported having a halo or glare following trifocal IOL implantation, but it was not considered bothersome in most cases^9^. However, the presence of halo and glare provided by most studies were reported subjectively without an objective evaluating system. Consequently, the conclusions of most studies were inconsistent or with inadequate methodology. In contrast with the previous meta-analysis^26^, our results showed a higher incidence of halos in trifocal IOLs. Trifocal IOLs, by design, were inevitably associated with 18% to 20% loss in light transmission, leading to a relatively blurred image^35^. Although such visual disturbance may hamper the wide acceptance of trifocal IOLs, such phenomena were considered acceptable in most studies and the assessment of photopsia relies largely on patients’ subjective perceptions and tolerance. With regard to subjective visual quality and visual function questionnaire, satisfactory postoperative visual quality and high level of patient satisfaction were achieved in both groups. Furthermore, optical compromise and a process of neuroadaptation by the patient is also necessary^3^. Therefore, the postoperative follow-up period is also relevant to how the patient adjusts to the retinal image.

To our knowledge, our meta-analysis provided the most up-to-date and comprehensive clinical indicators of trifocal and the hybrid multifocal-EDOF IOLs. However, several limitations should be noted. First, substantial heterogeneity was found between the studies. This might be due to the varied follow-up duration, different measurement standards and study locations and diverse patient characteristics. However, sensitivity analyses and subgroup analyses proved the stability of our results. Second, some of the RCTs and NRCSs indicated low quality, which may generate selection bias. Third, although the defocus curve offers detailed granularity of visual performance from different focal distances, insufficient data and different measurement techniques precluded meta-analysis of the defocus curve and contrast sensitivity. Consequently, more representative visual outcomes at different focal point were chosen and descriptive information was recorded.

In conclusion, the present meta-analysis demonstrated that the trifocal IOLs performed better than the hybrid multifocal-EDOF IOL at near distance but inevitably generated more photic effects in the form of halos. In real clinical practice, familiarity with IOLs characteristics helps to fulfill patients’ expectations and attain a high degree of satisfaction over time. However, in addition to IOL features, patients’ personalities, expectations, preoperative conditions and economic status should also be taken into account. More evidence-based publications and RCTs are warranted to provide guidelines for IOLs selection in order to provide maximum visual benefits and fulfill personalized visual needs in the future.

## Methods

The current meta-analysis was designed and performed based on the principles described in the Meta-Analysis of Observational Studies in Epidemiology (MOOSE) and the Preferred Reporting Items for Systematic Reviews and Meta-Analyses (PRISMA) guidelines^36,37^.

### Search strategy

We systematically searched the literature using the PubMed, EMBASE and Web of Science databases published through 1 March 2020. The following keywords were used: ("multifocal" OR "trifocal" OR "three foci") AND ("EDOF" OR "extended depth of focus") AND ("IOL" OR "intraocular lens"). The reference lists of the included articles and pertinent reviews were also searched.

### Eligibility criteria

We included all the RCTs and prospective cohorts comparing clinical outcomes between trifocal (FineVision Micro F IOL (PhysIOL Liege, Belgium), AT LISA tri 839MP IOL (Carl Zeiss Meditec AG, Jena, Germany), AcrySof IQ PanOptix IOL (Alcon Surgical, Inc., Fort Worth, TX)) and the TECNIS Symfony ZXR00 (Johnson & Johnson Vision, Santa Ana, California, USA) IOLs in patients who underwent bilateral cataract or RLE surgeries. Studies of patients with ocular diseases, such as corneal opacities, keratoconus, uveitis, macular disease and optic neuropathies were excluded. In addition, studies were excluded for double reporting, blended implantation and lack of bilateral data.

### Data collection and outcome variables

Two authors (Y. Z. and K.W.) independently screened all the titles and abstracts (where available) of all the studies identified in the database searches and then evaluated the full manuscript of the relevant articles. Any discrepancies were resolved through group discussion. The data of each eligible study were extracted in a standardized data collection form including the following baseline demographic and clinical data: first author, year of publication, country of origin, follow-up duration, type of intraocular lens, sample size, number of eyes, gender and age.

Primary outcomes comprised uncorrected and corrected VAs at near (40 cm), intermediate (60 cm) and far distances (4 m); spherical equivalent; spectacle independence at near, intermediate and far distances; and photic effects, such as halo or glare. VA was evaluated using the logMAR scale under photopic conditions. Participants who reported experiencing halo or glare were considered to have photic disturbance. Secondary outcomes included defocus curves, contrast sensitivity and visual quality questionnaires. Binocular defocus curves were measured under standard testing conditions with 0.50 D steps from + 1.00 to − 4.00 D. Contrast sensitivity values under photopic and scotopic conditions of 1.5, 3, 6, 12, and 18 cpd were included in the analysis. Since inadequate data on defocus curves and contrast sensitivity precluded further meta-analysis, descriptive information was provided instead.

### Quality assessment

Quality assessment of the RCTs and cohort studies were performed according to the Jaded scale and the NOS, respectively. The Jadad scale uses three primary aspects of randomization, blinding, and participant dropout. Appropriate randomization and blinding each scored two points, with total scores ranging from zero to five. Studies scoring more than three points were considered to be of high quality. The NOS assigns a maximum of nine points to each study: four points for the selection of participants and the measurement of exposure, two points for comparability, and three points for the assessment of outcomes and adequate follow-up. Studies with more than six points indicates high quality.

### Heterogeneity management

Forest plots were used to present the results, with lines representing the estimates from the different studies and their confidence intervals (CIs) and boxes graphically representing the weight given to each study in calculating the pooled estimate for a given outcome. Substantial heterogeneity was defined as *I*^2^ was > 50%, and the *P*-value for heterogeneity was *P* < 0.10. The fixed effect model was used when heterogeneity was small; otherwise, the random effects model was used^38–40^. Publication bias was evaluated using contour-enhanced funnel plots, the Egger linear regression test, and the Begg rank correlation test, with significance set to *P* < 0.10^41,42^. Sensitivity analyses were performed by omitting one study at a time and calculating a pooled estimate for the remainding of the studies. Subgroup analyses and meta-regression analyses, according to the type of IOL implanted and the follow-up duration, were performed to evaluate the source of the heterogeneity.

### Statistical analysis

All statistical analyses were performed using StataCorp. 2017. *Stata Statistical Software: Release 15*. College Station, TX: StataCorp LLC. Mean differences (MDs) and risk ratios (RRs)with 95% Cis were calculated for the continuous measures and dichotomous variables. Statistical significance was defined as *P* < 0.05.

## Supplementary Information

Supplementary Information
