# Supplementary material for: Comparison of trifocal or hybrid multifocal-extended depth of focus intraocular lenses: a systematic review and meta-analysis
Source: Sci Rep. 2021 Mar 23;11:6699. doi: 10.1038/s41598-021-86222-1 (PMC7987991; doi:10.1038/s41598-021-86222-1)
Supplement: Supplementary file 1 — Supplementary Information [file 41598_2021_86222_MOESM1_ESM.docx]

**Comparison of trifocal or** **hybrid multifocal-extended depth of focus intraocular lenses: a systematic review and meta-analysis**

Yueyang Zhong, MD ^1#^, Kai Wang, MD^1#^, Xiaoning Yu, MD^1^, Xin Liu, MD^1^, Ke Yao, MD^1^*

**Author affiliations:**

^1^Eye Center of the Second Affiliated Hospital, School of Medicine, Zhejiang University, Hangzhou, Zhejiang Province, China.

***Correspondence:** Ke YAO, MD, Professor and Chief. Eye Center, Second Affiliated Hospital, School of Medicine, Zhejiang University, No. 88 Jiefang Road, Hangzhou 310009, China. E-mail address: [xlren@zju.edu.cn](mailto:xlren@zju.edu.cn). Tel: +86-571-87783897. Fax: +86-571-87783897.

^#^ Yueyang Zhong and Kai Wang contributed equally to this work.

**Supplementary Information**

Supplementary Table S1. Jaded Scale for Randomized Controlled Trials (RCTs).

Supplementary Table S2. Newcastle–Ottawa Scale for Observational Studies (cohorts).

Supplementary Table S3. Sensitivity analyses by omitting a single study performed in uncorrected visual acuities.

Supplementary Table S4. Sensitivity analyses by omitting a single study performed in corrected visual acuities.

Supplementary Table S5. Sensitivity analyses by omitting a single study performed in spherical equivalent.

Supplementary Table S6. Descriptive information of questionnaires for visual quality.

Supplementary Table S7. Descriptive information of questionnaires for satisfaction.

Supplementary Table S8 Publication bias measured by Begg’s and Egger’s test.

Supplementary Fig. S1. Forest plots of visual acuities for intraocular lens types. Uncorrected distance visual acuity (a) and uncorrected near visual acuity (b).

Supplementary Fig. S2. Forest plots of spherical equivalent (SE) for intraocular lens types.

Supplementary Fig. S3. Forest plots of spherical equivalent (SE) excluding Singh (2019).

Supplementary Fig. S4. Meta regression for spherical equivalent (SE).

Supplementary Fig. S5. Funnel plots of included studies for visual acuities, spherical equivalent, spectacle independence and photic disturbance.

**Supplementary Table S1.** Jaded Scale for Randomized Controlled Trials (RCTs).

| **Author (year)** | **Randomization** | **Blinding** | **Withdraws** | **Overall quality** |
| --- | --- | --- | --- | --- |
| Monaco (2017)^1^ | 2 | 1 | 1 | 4 |
| Cochener (2018)^2^ | 1 | 0 | 1 | 2 |
| Gil (2020)^3^ | 2 | 1 | 1 | 4 |
| Webers (2020)^4^ | 2 | 2 | 1 | 5 |

**Average: 3.8**

**Supplementary Table S2.** Newcastle–Ottawa Scale for Observational Studies (cohorts).

| **Author (year)** | **Selection** | | | | **Comparability** | **Outcome** | | | **Overall quality** |
| --- | --- | --- | --- | --- | --- | --- | --- | --- | --- |
|  | **Representative of cases** | **Selection of controls** | **Exposure ascertainment** | **No history of disease** | **Comparable on confounders** | **Outcome assessment** | **Adequate follow-up time (> 3 months)** | **Follow-up rate (> 80%)** |  |
| Hamid and Sokwala (2016)^5^ | 0 | 1 | 1 | 1 | 2 | 1 | 1 | 1 | 8 |
| Ruiz-Mesa (2017)^6^ | 0 | 1 | 1 | 1 | 2 | 1 | 1 | 1 | 8 |
| Mencucci (2018)^7^ | 0 | 1 | 1 | 1 | 2 | 1 | 1 | 1 | 8 |
| Ruiz-Mesa (2018)^8^ | 0 | 1 | 1 | 1 | 2 | 1 | 1 | 1 | 8 |
| Böhm (2019)^9^ | 0 | 1 | 1 | 1 | 1 | 1 | 1 | 1 | 7 |
| de Medeiros (2019)^10^ | 0 | 1 | 1 | 1 | 2 | 1 | 1 | 1 | 8 |
| Escandon-Garcia (2019)^11^ | 0 | 1 | 1 | 1 | 2 | 1 | 0 | 1 | 7 |
| Rodov (2019)^12^ | 0 | 1 | 1 | 1 | 1 | 1 | 0 | 1 | 6 |
| Singh (2019)^13^ | 0 | 1 | 1 | 1 | 2 | 1 | 1 | 1 | 8 |
| **Average: 7.6** |  |  |  |  |  |  |  |  |  |

**Supplementary Table S3.** Sensitivity analyses by omitting a single study performed in uncorrected visual acuities.

| **Study excluded** | **UDVA** | | |  | **UIVA** | | |  | **UNVA** | | |  |
| --- | --- | --- | --- | --- | --- | --- | --- | --- | --- | --- | --- | --- |
|  | ***I*^2^ (%)** | ***P* for heterogeneity** | **MD (95% CI)** |  | ***I*^2^ (%)** | ***P* for heterogeneity** | **MD (95% CI)** |  | ***I*^2^ (%)** | ***P* for heterogeneity** | **MD (95% CI)** |  |
| Monaco (2017)^1^ | 67.50 | 0.001 | 0.02 (-0.00, 0.03) |  | 85.70 | < 0.001 | 0.07 (0.03, 0.10) |  | 85.10 | < 0.001 | -0.15 (-0.20, -0.11) |  |
| Ruiz-Mesa (2017)^6^ | 78.80 | < 0.001 | 0.01 (-0.01, 0.04) |  | 90.40 | < 0.001 | 0.06 (0.02, 0.10) |  | 91.70 | < 0.001 | -0.15 (-0.20, -0.10) |  |
| Cochener (2018)^2^ | 78.10 | < 0.001 | 0.01 (-0.01, 0.04) |  | 90.50 | < 0.001 | 0.05 (0.01, 0.10) |  | 91.90 | < 0.001 | -0.15 (-0.20- -0.10) |  |
| Escandon-Garcia (2018)^11^ | 79.10 | < 0.001 | 0.01 (-0.01, 0.03) |  | NA | NA | NA |  | NA | NA | NA |  |
| Mencucci (2018)^7^ | 79.10 | < 0.001 | 0.01 (-0.01, 0.03) |  | 90.20 | < 0.001 | 0.05 (0.01, 0.10) |  | 90.90 | < 0.001 | -0.14 (-0.20, -0.08) |  |
| Ruiz-Mesa (2018)^8^ | 77.60 | < 0.001 | 0.01 (-0.01, 0.03) |  | NA | NA | NA |  | NA | NA | NA |  |
| Böhm (2019)^9^ | 79.00 | < 0.001 | 0.01 (-0.01, 0.03) |  | 90.00 | < 0.001 | 0.05 (0.01, 0.10) |  | 90.10 | < 0.001 | -0.13 (-0.18, -0.08) |  |
| de Medeiros (2019)^10^ | 74.80 | < 0.001 | 0.00 (-0.01, 0.03) |  | 82.90 | < 0.001 | 0.04 (0.01, 0.07) |  | 91.80 | < 0.001 | -0.14 (-0.20, -0.09) |  |
| Rodov (2019)^12^ | 73.70 | < 0.001 | 0.01 (-0.01, 0.03) |  | 89.60 | < 0.001 | 0.06 (0.02, 0.11) |  | 89.60 | < 0.001 | -0.13 (-0.18, -0.08) |  |
| Gil (2020)^3^ | 75.50 | < 0.001 | 0.01 (-0.01, 0.02) |  | 89.10 | < 0.001 | 0.05 (0.01, 0.09) |  | 91.90 | < 0.001 | -0.14 (-0.19, -0.09) |  |
| Webers (2020)^4^ | 77.50 | < 0.001 | 0.01 (-0.01, 0.03) |  | 90.40 | < 0.001 | 0.06 (0.01, 0.11) |  | 90.10 | < 0.001 | -0.16 (-0.21, -0.11) |  |

UDVA, uncorrected distance visual acuity; UIVA, uncorrected intermediate visual acuity; UNVA, uncorrected near visual acuity; MD, mean difference; 95% CI, 95% confidence interval; NA, not available.

**Supplementary Table S4.** Sensitivity analyses by omitting a single study performed in corrected visual acuities.

| **Study excluded** | **CDVA** | | |  | **CIVA** | | |  | **CNVA** | | |  |
| --- | --- | --- | --- | --- | --- | --- | --- | --- | --- | --- | --- | --- |
|  | ***I*^2^ (%)** | ***P* for heterogeneity** | **MD (95% CI)** |  | ***I*^2^ (%)** | ***P* for heterogeneity** | **MD (95% CI)** |  | ***I*^2^ (%)** | ***P* for heterogeneity** | **MD (95% CI)** |  |
| Monaco (2017)^1^ | 66.00 | 0.012 | 0.01 (-0.01, 0.03) |  | 82.40 | 0.001 | 0.06 (0.01, 0.10) |  | 77.40 | 0.004 | -0.17 (-0.22, -0.13) |  |
| Escandon-Garcia (2018)^11^ | 60.90 | 0.026 | 0.01 (-0.01, 0.02) |  | NA | NA | NA |  | NA | NA | NA |  |
| Mencucci (2018)^7^ | 65.20 | 0.013 | 0.01 (-0.01, 0.02) |  | 91.10 | < 0.001 | 0.04 (-0.02, 0.10) |  | 94.70 | < 0.001 | -0.15 (-0.24, -0.06) |  |
| Ruiz-Mesa (2018)^8^ | 65.30 | 0.013 | 0.01 (-0.01, 0.03) |  | 90.80 | < 0.001 | 0.05 (-0.01, 0.11) |  | 94.40 | < 0.001 | -0.15 (-0.23, -0.06) |  |
| Böhm (2019)^9^ | 68.30 | 0.008 | 0.01 (-0.01, 0.03) |  | 90.30 | < 0.001 | 0.03 (-0.02, 0.09) |  | 87.80 | < 0.001 | -0.12 (-0.18, -0.07) |  |
| de Medeiros (2019)^10^ | 55.40 | 0.047 | 0.00 (-0.01, 0.02) |  | NA | NA | NA |  | NA | NA | NA |  |
| Gil (2020)^3^ | 54.50 | 0.052 | 0.01 (-0.01, 0.02) |  | 86.20 | < 0.001 | 0.02 (-0.02, 0.06) |  | 94.80 | < 0.001 | -0.15 (-0.23, -0.07) |  |

CDVA, corrected distance visual acuity; CIVA, corrected intermediate visual acuity; CNVA, corrected near visual acuity; MD, mean difference; 95% CI, 95% confidence interval; NA, not available.

**Supplementary Table S5.** Sensitivity analyses by omitting a single study performed in spherical equivalent.

| **Author (year)** | ***I*^2^ (%)** | ***P* for heterogeneity** | **MD (95% CI)** |
| --- | --- | --- | --- |
| Hamid and Sokwala (2016)^5^ | 92.40 | < 0.001 | -0.03 (-0.08, 0.02) |
| Monaco (2017)^1^ | 86.90 | < 0.001 | -0.04 (-0.11, 0.03) |
| Ruiz-Mesa (2017)^6^ | 93.50 | < 0.001 | -0.04 (-0.09, 0.01) |
| Cochener (2018)^2^ | 93.40 | < 0.001 | -0.03 (-0.09, 0.02) |
| Escandon-Garcia (2018)^11^ | 93.50 | < 0.001 | -0.05 (-0.10, 0.01) |
| Mencucci (2018)^7^ | 93.60 | < 0.001 | -0.04 (-0.10, 0.01) |
| Ruiz-Mesa (2018)^8^ | 92.40 | < 0.001 | -0.02 (-0.07, 0.03) |
| Böhm (2019)^9^ | 93.60 | < 0.001 | -0.04 (-0.10, 0.01) |
| de Medeiros (2019)^10^ | 93.60 | < 0.001 | -0.04 (-0.10, 0.01) |
| Rodov (2019)^12^ | 93.60 | < 0.001 | -0.05 (-010, 0.00) |
| Singh (2019)^13^ | 61.80 | 0.004 | -0.06 (-0.10, -0.01) |
| Webers (2020)^4^ | 93.50 | < 0.001 | -0.04 (-0.09, 0.01) |

MD, mean difference; 95% CI, 95% confidence interval.

**Supplementary Table S6. Descriptive information of questionnaires for visual quality.**

| **Author (year)** | **Trifocal IOLs** | **MF/EDOF IOL** | **Results of visual quality** |
| --- | --- | --- | --- |
| Monaco (2017)^1^ | PanOptix | Symfony | No significant differences in the QoV questionnaire (*P*=0.32). |
| Cochener (2018)^2^ | PanOptix  FineVision | Symfony | No significant differences in the QoV questionnaire. |
| Escandón-García (2018)^11^ | PanOptix  FineVision | Symfony | Symfony IOL presented worse performance in the bothersome subscale of the QoV questionnaire (*P*=0.011). |
| de Medeiros (2019)^10^ | PanOptix | Symfony | No significant differences in the NEI-VFQ 25 questionnaire. |
| Singh (2019)^13^ | FineVision | Symfony | No significant differences in the QoV questionnaire (*P*=0.32). |
| Gil (2020)^3^ | AT LISA tri 839MP | Symfony | Symfony IOL presented better performance in VF-14 questionnaire (*P*=0.039). |
| Webers (2020)^4^ | AT LISA tri 839MP | Symfony | No significant differences in the VQOL questionnaire (*P*=0.81). |

IOL, intraocular lens; MF, multifocal; EDOF, extended depth of focus; NR, not reported; QoV, quality of vision; NEI-VFQ, National Eye Institute Visual Functioning Questionnaire; VF-14, visual functioning questionnaire-14; VQOL, vision-related quality of life.

**Supplementary Table S7. Descriptive information of questionnaires for satisfaction.**

| **Author (year)** | **Trifocal IOLs** | **MF/EDOF IOL** | **Results of patient satisfaction** |
| --- | --- | --- | --- |
| Hamid and Sokwala (2016)^5^ | FineVision  AT LISA tri 839MP | Symfony | High patient satisfaction (93%–94%) with Symfony and AT LISA tri 839, and 80% with FineVision. |
| Ruiz-Mesa (2017)^6^ | FineVision | Symfony | 80% satisfied in trifocal group and 90% in Symfony group. 95% would undergo implantation of the same IOL again. |
| Cochener (2018)^2^ | PanOptix  FineVision | Symfony | All patients stated that they would be willing to repeat surgery with the same IOL. |
| Mencucci (2018)^7^ | PanOptix  AT LISA tri 839MP | Symfony | All patients were fully satisfied and would have chosen the same lens again. |
|  |  |  |  |
| Rodov (2019)^12^ | FineVision | Symfony | 78% for the FineVision and 76% for the Symfony reported that they would choose the same IOL again. |
| Webers (2020)^4^ | AT LISA tri 839MP | Symfony | 92% and 93% of patients for the AT LISA tri 839 and Symfony groups would recommend the implanted IOL to family or friends. |

IOL, intraocular lens; MF, multifocal; EDOF, extended depth of focus; NR, not reported.

**Supplementary Table S8** Publication bias measured by Begg’s and Egger’s test.

| **Subject** | | **Begg's test** | **Egger's test** |
| --- | --- | --- | --- |
| **Visual acuity** | |  |  |
|  | UDVA | 1.000 | 0.670 |
|  | UIVA | 0.251 | 0.208 |
|  | UNVA | 1.000 | 0.657 |
|  | CDVA | 0.764 | 0.450 |
|  | CIVA | 0.086 | 0.193 |
|  | CNVA | 0.462 | 0.155 |
| **UIVA by IOL types** | |  |  |
|  | AT LISA tri 839MP | 0.296 | 0.369 |
|  | PanOptix | 0.308 | 0.153 |
|  | FineVision | 1.000 | 0.330 |
| **Spherical equivalent** | | 0.150 | 0.160 |
| **Spectacle independence** | |  |  |
|  | Far distance | 0.296 | 0.164 |
|  | Intermediate distance | NA | NA |
|  | Near distance | 0.260 | 0.163 |
| **Photic disturbance** | |  |  |
|  | halo | 0.462 | 0.317 |
|  | glare | 1.000 | 0.455 |

UDVA, uncorrected distance visual acuity; UIVA, uncorrected intermediate visual acuity; UNVA, uncorrected near visual acuity; CDVA, corrected distance visual acuity CIVA, corrected intermediate visual acuity; CNVA, corrected near visual acuity; IOL, intraocular lens; NA, not available.

**Supplementary Fig. S1.** Forest plots of visual acuities for intraocular lens types. Uncorrected distance visual acuity (a) and uncorrected near visual acuity (b).


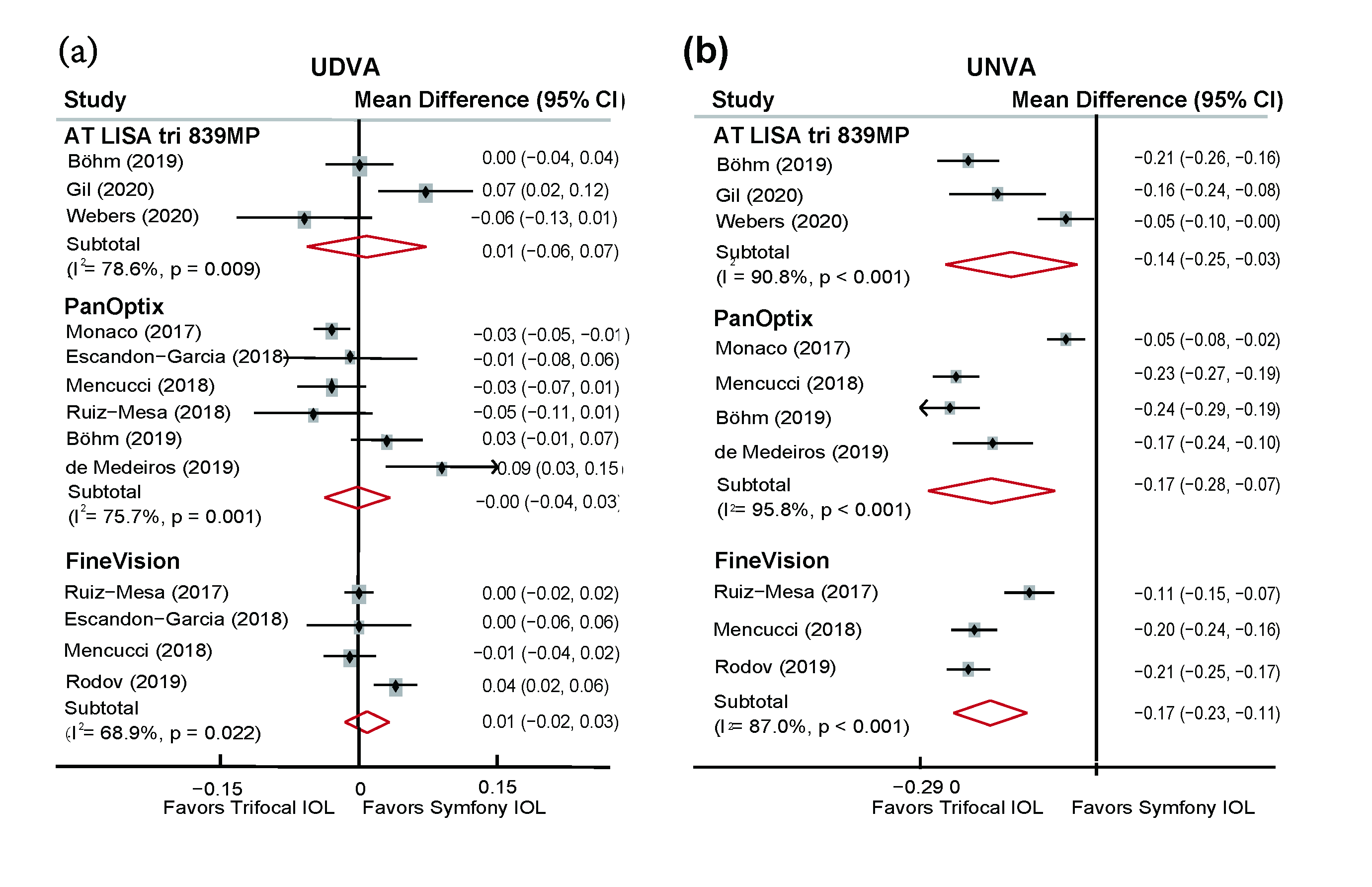


**Supplementary Fig. S2.** Forest plots of spherical equivalent (SE) for intraocular lens types.


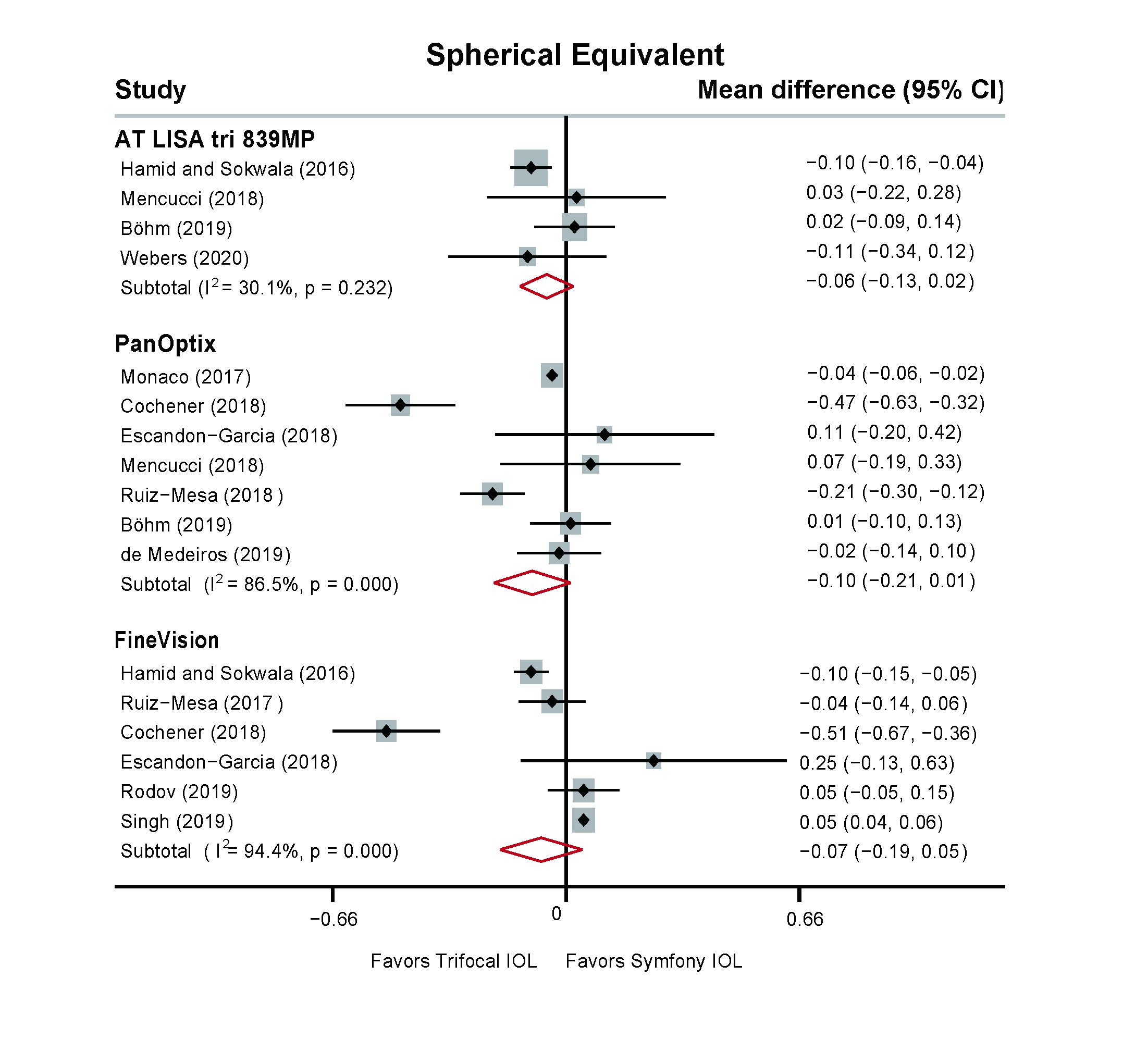


**Supplementary Fig. S3.** Forest plots of spherical equivalent (SE) excluding Singh (2019).


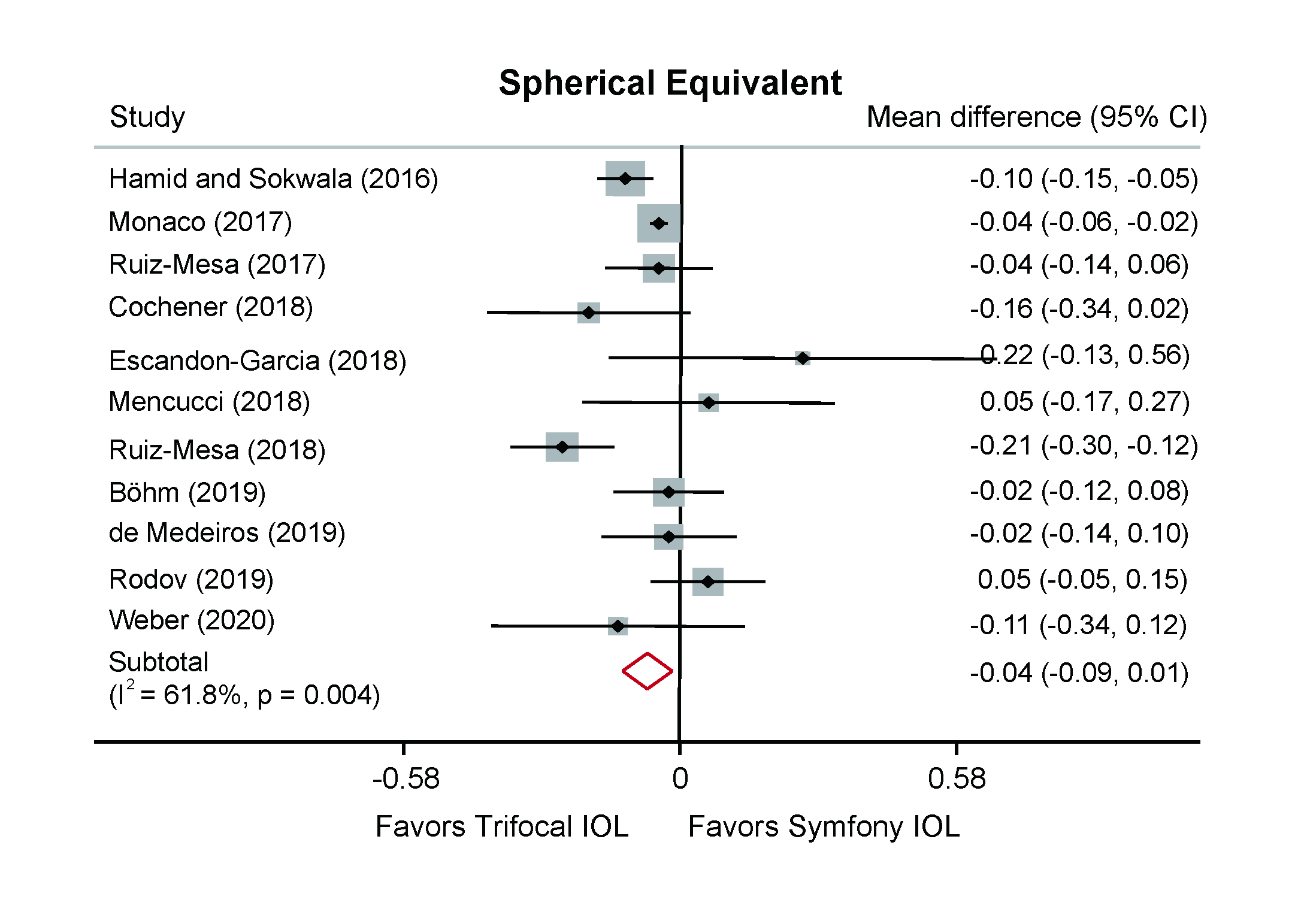


**Supplementary Fig. S4.** Meta regression for spherical equivalent (SE).


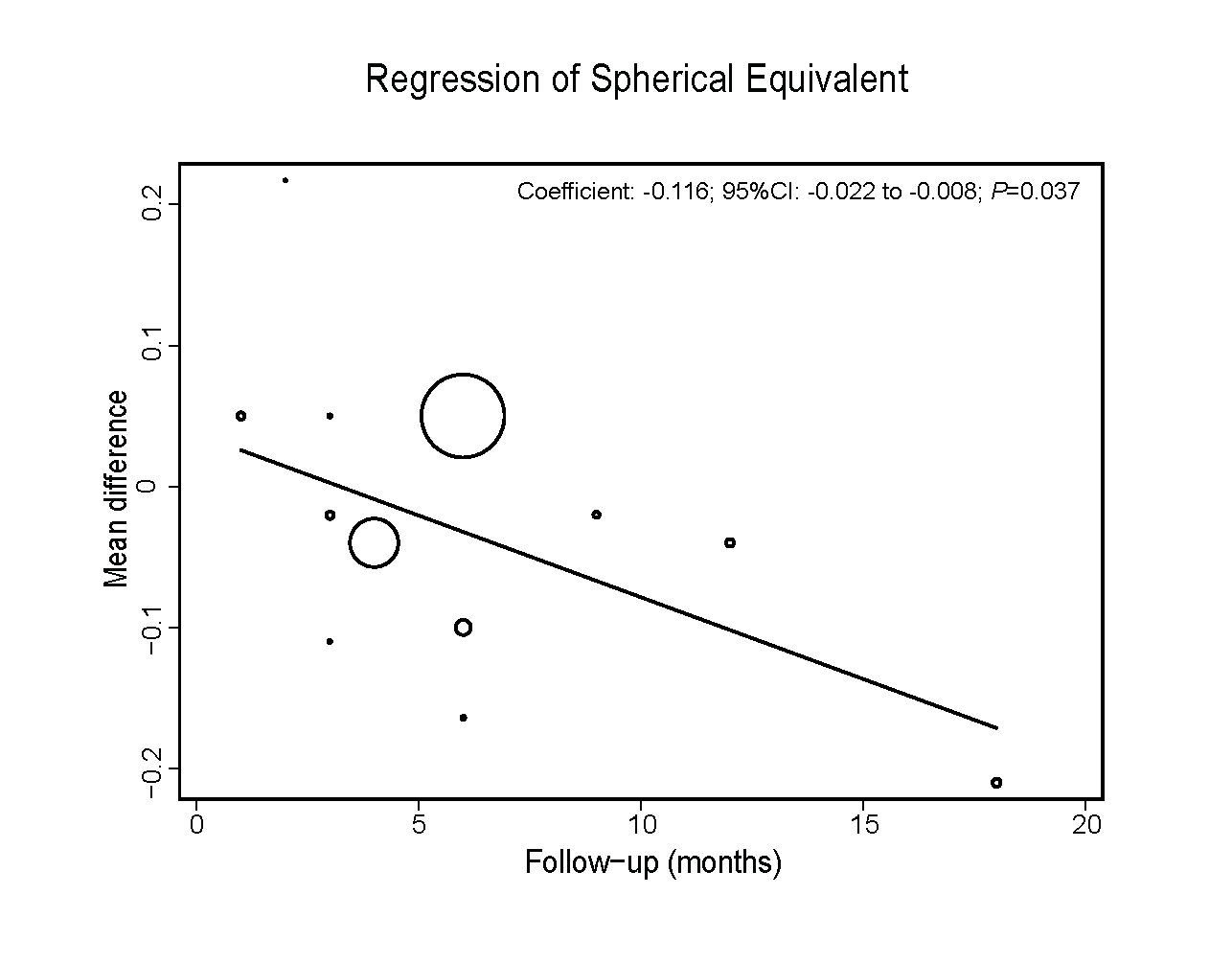


**Supplementary Fig. S5.** Funnel plots of included studies for visual acuities, spherical equivalent, spectacle independence and photic disturbance.


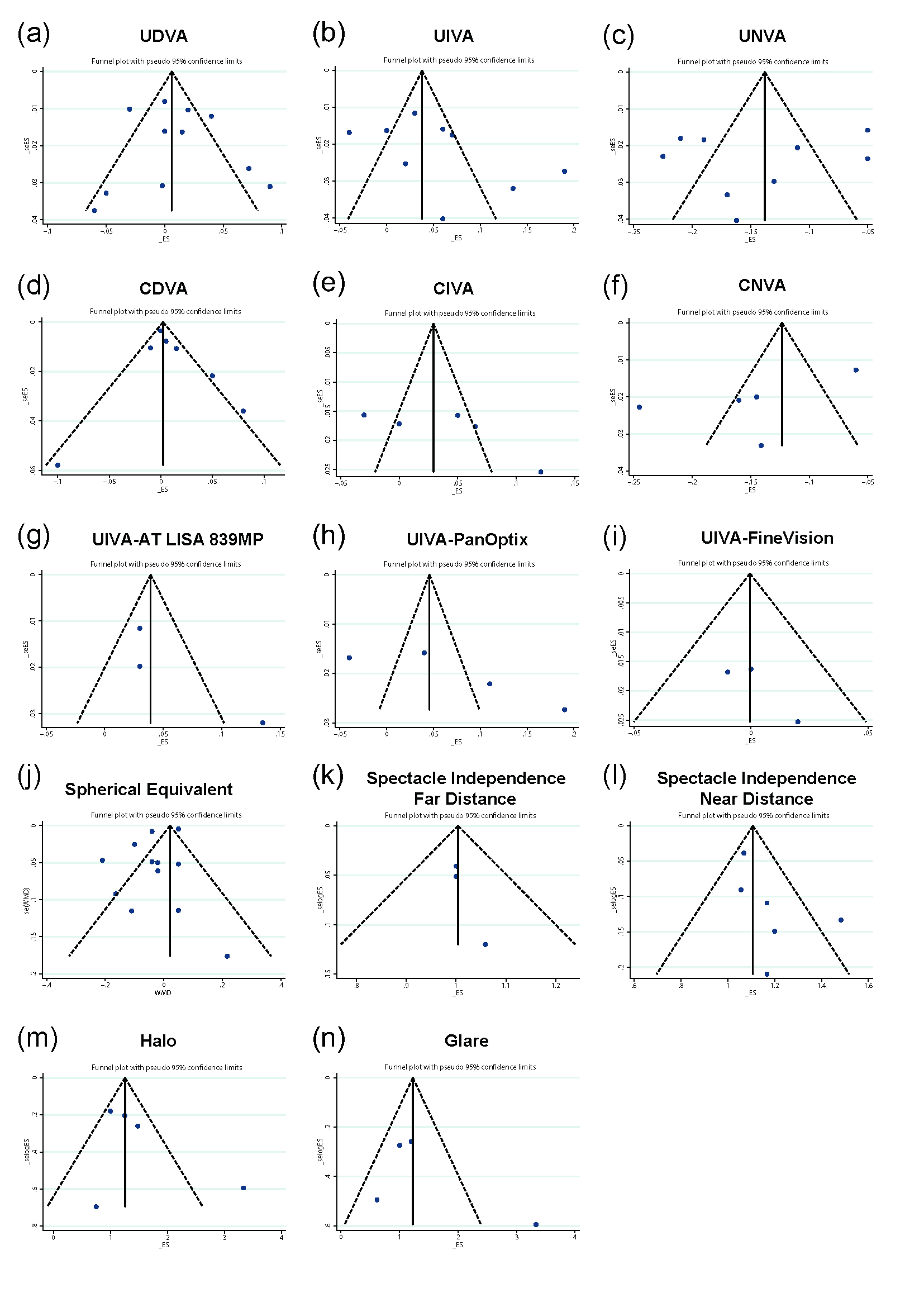


**References**

1. Monaco, G. *et al.* Visual performance after bilateral implantation of 2 new presbyopia-correcting intraocular lenses: Trifocal versus extended range of vision: *Journal of Cataract & Refractive Surgery* **43**, 737–747 (2017).

2. Cochener, B., Boutillier, G., Lamard, M. & Auberger-Zagnoli, C. A Comparative Evaluation of a New Generation of Diffractive Trifocal and Extended Depth of Focus Intraocular Lenses. *Journal of Refractive Surgery* **34**, 507–514 (2018).

3. Gil, M. A., Varón, C., Cardona, G. & Buil, J. A. Visual acuity and defocus curves with six multifocal intraocular lenses. *Int Ophthalmol* **40**, 393–401 (2020).

4. Webers, V. S. C. *et al.* Comparison of the intermediate distance of a trifocal IOL with an extended depth-of-focus IOL: results of a prospective randomized trial. *J Cataract Refract Surg* **46**, 193–203 (2020).

5. Hamid, A. & Sokwala, A. A More Natural Way of Seeing: Visual Performance of Three Presbyopia Correcting Intraocular Lenses. *Open Journal of Ophthalmology* **06**, 176–183 (2016).

6. Ruiz-Mesa, R., Abengózar-Vela, A., Aramburu, A. & Ruiz-Santos, M. Comparison of Visual Outcomes after Bilateral Implantation of Extended Range of Vision and Trifocal Intraocular Lenses. *European Journal of Ophthalmology* **27**, 460–465 (2017).

7. Mencucci, R., Favuzza, E., Caporossi, O., Savastano, A. & Rizzo, S. Comparative analysis of visual outcomes, reading skills, contrast sensitivity, and patient satisfaction with two models of trifocal diffractive intraocular lenses and an extended range of vision intraocular lens. *Graefe’s Archive for Clinical and Experimental Ophthalmology* **256**, 1913–1922 (2018).

8. Ruiz-Mesa. A comparative study of the visual outcomes between a new trifocal and an extended depth of focus intraocular lens. *European journal of ophthalmology* **28**, 182‐187 (2018).

9. Böhm M., Petermann K., Hemkeppler E. & Kohnen T. Defocus curves of 4 presbyopia-correcting IOL designs: Diffractive panfocal, diffractive trifocal, segmental refractive, and extended-depth-of-focus. *J. Cataract Refractive Surg.* **45**, 1625–1636 (2019).

10. de Medeiros, A. L. *et al.* Comparison of visual outcomes after bilateral implantation of two intraocular lenses with distinct diffractive optics. *Clinical Ophthalmology* **Volume 13**, 1657–1663 (2019).

11. Escandón-García, S., Ribeiro, F. J., McAlinden, C., Queirós, A. & González-Méijome, J. M. Through-Focus Vision Performance and Light Disturbances of 3 New Intraocular Lenses for Presbyopia Correction. *Journal of Ophthalmology* **2018**, 1–8 (2018).

12. Rodov, L., Reitblat, O., Levy, A., Assia, E. I. & Kleinmann, G. Visual Outcomes and Patient Satisfaction for Trifocal, Extended Depth of Focus and Monofocal Intraocular Lenses. *Journal of Refractive Surgery* **35**, 434–440 (2019).

13. Singh, B., Sharma, S., Dadia, S., Bharti, N. & Bharti, S. Comparative Evaluation of Visual Outcomes After Bilateral Implantation of a Diffractive Trifocal Intraocular Lens and an Extended Depth of Focus Intraocular Lens: *Eye & Contact Lens: Science & Clinical Practice* 1 (2019) doi:10.1097/ICL.0000000000000637.
